# Development and Optimization of a Machine-Learning Prediction Model for Acute Desquamation After Breast Radiation Therapy in the Multicenter REQUITE Cohort

**DOI:** 10.1016/j.adro.2021.100890

**Published:** 2022-01-03

**Authors:** Mahmoud Aldraimli, Sarah Osman, Diana Grishchuck, Samuel Ingram, Robert Lyon, Anil Mistry, Jorge Oliveira, Robert Samuel, Leila E.A. Shelley, Daniele Soria, Miriam V. Dwek, Miguel E. Aguado-Barrera, David Azria, Jenny Chang-Claude, Alison Dunning, Alexandra Giraldo, Sheryl Green, Sara Gutiérrez-Enríquez, Carsten Herskind, Hans van Hulle, Maarten Lambrecht, Laura Lozza, Tiziana Rancati, Victoria Reyes, Barry S. Rosenstein, Dirk de Ruysscher, Maria C. de Santis, Petra Seibold, Elena Sperk, R. Paul Symonds, Hilary Stobart, Begoña Taboada-Valadares, Christopher J. Talbot, Vincent J.L. Vakaet, Ana Vega, Liv Veldeman, Marlon R. Veldwijk, Adam Webb, Caroline Weltens, Catharine M. West, Thierry J. Chaussalet, Tim Rattay, REQUITE consortium

**Affiliations:** a Health Innovation Ecosystem, University of Westminster, London, United Kingdom; b Patrick G. Johnston Centre for Cancer Research, Queen's University Belfast, Belfast, United Kingdom; c Imperial College Healthcare NHS Trust, London, United Kingdom; d Division of Cancer Sciences, Faculty of Biology, Medicine and Health, University of Manchester, Manchester, United Kingdom; e Department of Computer Science, Edge Hill University, Ormskirk, Lancashire, United Kingdom; f Guy's and St. Thomas’ NHS Foundation Trust, London, United Kingdom; g Mirada Medical, Oxford, United Kingdom; h University of Leeds, Leeds Cancer Centre, St. James's University Hospital, Leeds, United Kingdom; i Edinburgh Cancer Centre, Western General Hospital, Edinburgh, United Kingdom; j School of Computing, University of Kent, Canterbury, United Kingdom; k School of Life Sciences, University of Westminster, London, United Kingdom; l Fundación Publica Galega Medicina Xenomica, Santiago de Compostela, Spain; m Instituto de Investigación Sanitaria de Santiago (IDIS), Servicio Galego de Saúde (SERGAS), Santiago de Compostela, Spain; n University of Montpellier, Montpellier, France; o Division of Cancer Epidemiology, German Cancer Research Center (DKFZ), Heidelberg, Germany; p UKE University Cancer Center Hamburg, University Medical Center Hamburg-Eppendorf, Hamburg, Germany; q Centre for Cancer Genetic Epidemiology, University of Cambridge, Strangeways Research Laboratory, Worts Causeway, Cambridge, United Kingdom; r Radiation Oncology Department, Vall d'Hebron Hospital Universitari, Vall d'Hebron Hospital Campus, Barcelona, Spain; s Department of Radiation Oncology, Icahn School of Medicine at Mount Sinai, New York, New York; t Hereditary Cancer Genetics Group, Vall d'Hebron Institute of Oncology (VHIO), Vall d'Hebron Hospital Campus, Barcelona, Spain; u Department of Radiation Oncology, Universitätsmedizin Mannheim, Medical Faculty Mannheim, Heidelberg University, Mannheim, Germany; v Department of Human Structure and Repair, Ghent University, Ghent, Belgium; w Department of Radiation Oncology, University Hospital, Leuven, Belgium; x Department of Radiation Oncology 1, Fondazione IRCCS Istituto Nazionale dei Tumori, Milan, Italy; y Prostate Cancer Program, Fondazione IRCCS Istituto Nazionale dei Tumori, Milan, Italy; z Icahn School of Medicine at Mount Sinai, New York, New York; aa Maastricht University Medical Center, Department of Radiation Oncology (Maastro), GROW, Maastricht, The Netherlands; bb Cancer Research Centre, University of Leicester, Leicester, United Kingdom; cc Independent Cancer Patients’ Voice, London, United Kingdom; dd Department of Radiation Oncology, Complexo Hospitalario Universitario de Santiago, Servicio Galego de Saúde (SERGAS), Santiago de Compostela, Spain; ee Department of Genetics and Genome Biology, University of Leicester, Leicester, United Kingdom; ff Biomedical Network on Rare Diseases (CIBERER), Madrid, Spain; gg University of Manchester, Christie Hospital, Manchester, United Kingdom

## Abstract

**Purpose:**

Some patients with breast cancer treated by surgery and radiation therapy experience clinically significant toxicity, which may adversely affect cosmesis and quality of life. There is a paucity of validated clinical prediction models for radiation toxicity. We used machine learning (ML) algorithms to develop and optimise a clinical prediction model for acute breast desquamation after whole breast external beam radiation therapy in the prospective multicenter REQUITE cohort study.

**Methods and Materials:**

Using demographic and treatment-related features (m = 122) from patients (n = 2058) at 26 centers, we trained 8 ML algorithms with 10-fold cross-validation in a 50:50 random-split data set with class stratification to predict acute breast desquamation. Based on performance in the validation data set, the logistic model tree, random forest, and naïve Bayes models were taken forward to cost-sensitive learning optimisation.

**Results:**

One hundred and ninety-two patients experienced acute desquamation. Resampling and cost-sensitive learning optimisation facilitated an improvement in classification performance. Based on maximising sensitivity (true positives), the “hero” model was the cost-sensitive random forest algorithm with a false-negative: false-positive misclassification penalty of 90:1 containing m = 114 predictive features. Model sensitivity and specificity were 0.77 and 0.66, respectively, with an area under the curve of 0.77 in the validation cohort.

**Conclusions:**

ML algorithms with resampling and cost-sensitive learning generated clinically valid prediction models for acute desquamation using patient demographic and treatment features. Further external validation and inclusion of genomic markers in ML prediction models are worthwhile, to identify patients at increased risk of toxicity who may benefit from supportive intervention or even a change in treatment plan.

## Introduction

Radiation therapy is recommended for all patients with breast cancer who have a local excision and after mastectomy in high-risk patients.^1^ Over 70% of patients with breast cancer receive radiation therapy, which reduces local recurrence rates and increases long-term survival.^2^ As survival from breast cancer continues to improve,^3^ quality of life and survivorship have become increasingly important research priorities.^4^ Risk of radiation toxicity can be estimated from empirical dosimetric models based on the dose to the target organ and surrounding tissue.^5^ However, there is considerable variation between individual patient normal tissue reaction to radiation therapy and the extent to which they develop toxicity.^6^ Acute toxicity (<90 days from starting treatment) includes breast erythema and desquamation (skin loss).^7^ In a minority of patients, desquamation can cause substantial patient morbidity, worsen the cosmetic outcome after surgery, and affect quality of life.^8^ It can even result in the interruption of radiation therapy or a dose reduction, potentially increasing the risk of local recurrence.

Several studies have examined the association between acute breast radiation toxicity and clinical or treatment risk factors.^9^, ^10^, ^11^, ^12^, ^13^, ^14^, ^15^, ^16^, ^17^, ^18^ Nevertheless, statistical models have had limited success to date in predicting individual patient toxicity risk,^19^ and there is a paucity of validated prediction models for acute breast radiation toxicity. It is hypothesized that earlier prediction models failed to validate because they did not include sufficient variables to capture the variety of scenarios that occur among individual patients and individual treatment settings. Recent studies have demonstrated the capability of machine learning (ML) to develop predictive models for radiation toxicities in different cancers,^20^^,^^21^ including a thermal image-based random forest (RF) classifier for radiation dermatitis (skin erythema) after the first week of radiation therapy.^22^ Another recently published abstract describes how RF, gradient boosted decision tree, and logistic regression models were trained and validated on treatment planning and patient data comprising 230 variables including toxicity symptoms from patients at 5 collaborating U.S. centers to predict moist desquamation and Common Terminology Criteria for Adverse Events (CTCAE) grade ≥2 radiation dermatitis.^23^

For cancers with generally good local tumor control such as breast cancer, it is hypothesized that if a patient's individual risk of radiation toxicity could be estimated at the time of diagnosis, this could inform discussions about risks and benefits and allow treatment plans to be personalized for high-risk patients to minimize toxicity. Clinicians are particularly interested in models that include readily available clinical and treatment variables, which would allow toxicity risk to be estimated before treatment is planned. It is also important to predict toxicities that are sufficiently significant to warrant increased supportive intervention or treatment de-escalation. To that extent, a logistic regression model for acute breast desquamation after external beam radiation therapy (EBRT) recently developed in 3 combined radiation therapy cohorts failed to validate externally in the multicenter international REQUITE cohort.^24^ Therefore, the aim of this study was to use ML algorithms to develop and optimise a prediction model for acute breast desquamation after EBRT in the REQUITE breast cancer cohort.

## Methods and Materials

**Fig. 1:**
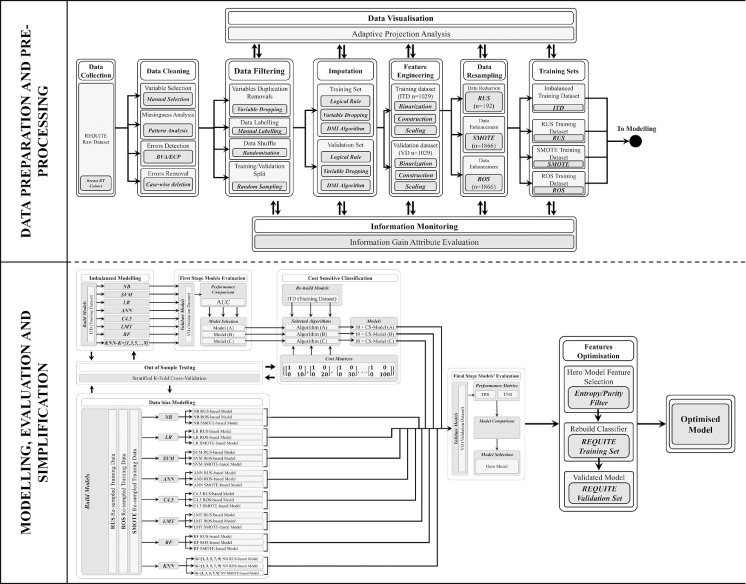
Diagram depicting the overall study design showing data preprocessing, splitting, and imputation at the top, and model development and optimization at the bottom. Abbreviations: ANN = artificial neural network; BVA = boundary value analysis; C4.5 = C4.5 decision tree; CS = cost sensitive optimisation; DMI = decision-tree-based missing-value imputation; ECP = equivalence class partitioning; ITD = imbalanced training data set; KNN = K-nearest neighbor; LMT = logistic model tree; LR = logistic regression; NB = naïve Bayes; RF = random forest; ROS = random over-sampling; RUS = random under-sampling; SMOTE = synthetic minority oversampling technique; SVM = support vector mechanism; VD = validation data set.

This was a TRIPOD (transparent reporting of a multivariable prediction model for individual prognosis or diagnosis) type 2a study using a single data set with a random split sample for development and validation.^25^ The full study design is shown in Figure 1.

### Study cohort and participants

REQUITE is an international, prospective cohort study that recruited patients with cancer before radiation therapy in 26 hospitals from 8 countries between April 2014 and March 2017 with unified standardized data collection.^26^ Patient baseline characteristics and methodology have been described in detail elsewhere.^27^ The present study used data from the breast cancer cohort (n = 2069). All patients were treated with breast-conserving surgery followed by whole breast EBRT according to local protocol. Partial breast irradiation and brachytherapy were excluded. Patients were assessed at the start and end of radiation therapy, and annually thereafter. Data collected at the start and at the end of radiation treatment were used to document acute toxicity. All patients gave written informed consent. The study was approved by local ethics committees in participating countries and registered at www.controlled-trials.com (ISRCTN 98496463).

### Endpoint definition

Toxicity in REQUITE was scored by treating physicians using CTCAE v4.0.^28^ CTCAE v4.0 has separate scales for radiation dermatitis (erythema) and skin ulceration (skin loss). The primary endpoint of this study was acute desquamation (skin loss or moist desquamation) occurring by the end of radiation therapy, defined as either CTCAE grade ≥3 radiation dermatitis (moist desquamation) or CTCAE grade ≥1 skin ulceration, implying that skin integrity was broken over the breast or in the inframammary fold. Patients with high baseline scores (defined as CTCAE grade >1 radiation dermatitis or CTCAE grade >0 skin ulceration) were excluded from the analysis, as this would not be attributable to the effect of radiation therapy.

### Variable selection, imputation, and preprocessing

The raw REQUITE data set (n = 2069) contained m = 204 variables (features) relating to patient baseline characteristics, comorbidities, cotreatments, and radiation therapy. Variables were initially checked for plausibility using domain expertise by physicians and radiation therapy physicists, and m = 136 variables remained. Boundary value analysis and equivalence class partitioning techniques^29^ were used for correcting or removing corrupt or inaccurate records from the data set. After variable-dropping (m = 13 with >37% missing values at random compared with observed values in the remaining variables) and case-wise deletion (n = 11 with missing class endpoint observations),^30^ the final data set for modeling had m = 123 variables including the endpoint variable and n = 2058 patient records.

The final data set was randomly shuffled and split 50:50 into training and test sets with class stratification, yielding imbalanced training (ITD, n = 1029) and validation (VD, n = 1029) data sets. Each data set was imputed independently with the ML decision-tree-based missing-value imputation technique^31^ to enhance best expectations of missing values. By carrying out the imputation of the data sets separately, the ITD and VD remained completely independent and perfectly isolated. Information levels were monitored in each data set pre- and postimputation with information gain attribute evaluation^32^ (see Fig. E4). The information gain of a feature is defined as the expected reduction of entropy (uncertainty within the data set) when partitioning the data; in other words, by how much the prediction of the endpoint/class would improve if the data were split using just that feature. The more plausible the pattern of information gain among data sets, the less bias is introduced in modeling. The evaluation of information worth is affected by the number of records and the 50:50 training-test split allowed for a fair information bias comparison between training and validation data sets.

The final set of m = 123 features consisted of 106 raw variables. Sixteen additional features were constructed to account for the vast number of possible combinations of chemotherapeutic agents received by some patients before radiation therapy. The adjuvant chemotherapy regimens were binarized based on their generic drug names (not shown). To adjust for different radiation therapy regimens, dose was calculated as the biologically effective dose (BED). BED is the product of the number of fractions (n), dose per fraction (d), and a factor determined by the dose and α/β ratio (10 Gy) for desquamation (acute toxicity):

$$BED=n\;d\;\left(1\;+\frac{\;d\;}{\alpha /\beta }\right)$$

The endpoint definition (acute desquamation = Desq) was used to label the patients to create a binary class variable. All numeric features (m = 63) were normalised with z score standardization.^33^

### Resampling

Although it is a clinically significant side effect from breast radiation therapy, only a small proportion of patients suffer from acute desquamation, an issue known as “class imbalance.”^9^^,^^18^ Both ITD and VD in this study were equally imbalanced ($Desq^{+}=\;96,\;Desq^{-}=\;933$). To address the issue of class imbalance, 3 resampling techniques were applied to the training data to obtain equal proportions of records in each class: random under-sampling (RUS) (n = 192, $\text{Des}q^{+}=\;96,\;\text{Des}q^{-}=\;96$),^34^ random over-sampling (ROS) ($n\;=\;1866,\;\text{Des}q^{+}=\;933,\;\text{Des}q^{-}=\;933$),^35^ and the synthetic minority oversampling technique (SMOTE) ($n\;=\;1866,\;\text{Des}q^{+}=\;933,\;\text{Des}q^{-}=\;933$).^36^ The effect of resampling techniques on the training data set was monitored with a multidimensional adaptive projection analysis into a 3-dimensional point cloud. Adaptive projection analysis^37^ is a multidimensional tool to visualise the classes that can be separated, any outliers or sources of error in the classification algorithms, and the existence of clusters in the data (see Fig. E5).

### Modeling

Eight different ML algorithms were used to build binary classification models to predict acute desquamation in patients undergoing breast-conserving surgery and adjuvant whole breast EBRT. They were trained in the ITD (imbalanced modeling, Fig. 1) as well as in the 3 resampled data sets (RUS, ROS, SMOTE; data-bias modeling, Fig. 1) with 10-fold cross-validation to reduce overfitting,^38^ and then each was tested in the VD (see Fig. 1). The ML alogrithms were discretized naïve Bayes (NB), logistic regression with ridge estimator,^39^ artificial neural networks with a multilayer perceptron architecture,^40^ support vector machine with polynomial kernel and logistic calibrator, K-nearest neighbour^41^ with K = 1,3,5,7,9, decision trees (C4.5),^32^ logistic model tree (LMT),^42^ and RF.^43^

Model performance was assessed using the area under the curve (AUC). The models with the highest AUC in the VD were taken forward for cost-sensitive learning optimisation. Cost-sensitive classification addresses the issue of class imbalance by imposing penalties (costs) for the misclassification of the positive cases (ie, making a false negative [FN] prediction). In this study, the cost for a FN prediction was not linked to a monetary value, instead a 10-step incremental inverse class distribution cost was used.^44^ The ITD has a 96:933 ≅ 1:10 ratio of examples in the positive class to examples in the negative class. This ratio is inverted to penalize FN with a 10-step incrementation at an initial cost x:1 of 10:1 increasing to 100:1. The cost is applied in the form of Charles Elkan's explicit cost matrix notation^45^:

$$Cost\;Matrix\;combinations\left[\begin{array}{cc} \text{FP}\left(1\right) & \text{TN}\left(0\right)\\ \text{TP}\left(0\right) & \text{FN}\left(x\right) \end{array}\right]=\left\{\left[\begin{array}{cc} 1 & 0\\ 0 & 10 \end{array}\right],\left[\begin{array}{cc} 1 & 0\\ 0 & 20 \end{array}\right],\left[\begin{array}{cc} 1 & 0\\ 0 & 30 \end{array}\right],\cdots ,\left[\begin{array}{cc} 1 & 0\\ 0 & 100 \end{array}\right]\right\}$$

AUC, sensitivity (true positive rate [TPR]), and specificity (true negative rate [TNR]) were used to compare and interpret the final models’ performance including those developed in the resampled data sets (see bottom half of Fig. 1). Final model selection was based on performance in the VD in terms of AUC and the clinicians’ trade-off maximizing both TPR and TNR. The selected model was further optimized using the mean decrease impurity entropy filter to select fewer features and simplify the “hero” model.^46^ All ML algorithms were implemented in the Waikato environment for knowledge analysis 3.8.3 (with the default models' parameters settings),^47^ with the C4.5 decision tree using the J48^48^ implementation, K-nearest neighbor using the IBK (instance-bases learning with parameter k) implementation, and support vector machine using the SMO (sequential minimal optimization)^49^ implementation.

## Results

Table 1 shows the main patient and treatment demographics for eligible patients. Median patient age was 58 years (range, 23-80 years). Patients were treated with a median breast dose of 50 Gy (28.5-56 Gy) in 25 fractions (range, 5-31) according to local protocol. In terms of important demographic features, 54.0% of patients had a body mass index ≥25, 42.7% were previous or current smokers, 31.0% had also undergone chemotherapy, 6.1% had diabetes, and 28.0% and 6.9% had hypertension and cardiovascular disease, respectively. About half of the patients were treated with intensity modulated radiation therapy, with a lower proportion in France and none at Italian or U.S. centers. The majority of patients received a tumor-bed boost (64%), ranging from less than 20% at the French, Italian, and Spanish centers to over 80% at the Belgian centers, given either simultaneously (n = 257) or sequentially (n = 1138). Patients with invasive breast cancer in Belgium and the United Kingdom were treated using the Standardisation of Breast Radiation therapy Trial B (START-B) hypofractionated regimen (40 Gy in 15 fractions). In terms of regional nodal irradiation, axillary nodes were treated in 11.9% and the supraclavicular fossa was treated in 12.8% of patients, respectively. Detailed characteristics of the REQUITE patient cohorts have previously been described elsewhere.^27^

**Table 1:** Summary study characteristics of eligible patients from the REQUITE patient cohort

	REQUITE breast cancer cohort
Eligible patients	2059
Location	Western Europe, United States
Study design	Prospective cohort
Recruitment year (range)	2014-2016
Treatment year (range)	2014-2016
Toxicity assessment scale	CTCAE v4.0
Toxicity assessment time points	Start-of-RT
	End-of-RT
Age (median, range)	58 (23-90)
Whole breast dose (Gy, median, range)	50 (28.5-56)
Whole breast fractions (median, range)	25 (5-31)
Hypofractionated regimen (proportion of patients)	47.9%
IMRT, simple field-in-field	39.7%
IMRT, complex modulated	9.8%
RT to axilla	11.9%
RT to supraclavicular fossa	12.8%
Boost	67.8%
BMI ≥25	54.0%
Smoker (current or previous)	42.7%
Chemotherapy	31.0%
Diabetes	6.1%
Hypertension	28.0%
Cardiovascular disease	6.9%
Toxicity (end of treatment)	
Ulceration	
Grade 0	1868 (91.2%)
Grade ≥1	181 (8.8%)
Dermatitis	
Grade 0	257 (12.5%)
Grade 1	1288 (62.6%)
Grade 2	462 (22.4%)
Grade 3	28 (1.4%)
Acute desquamation	
Ulceration ≥G1 or dermatitis ≥G3	192 (9.3%)

Abbreviations: BMI = body mass index; CTCAE = Common Terminology Criteria for Adverse Events; IMRT = intensity modulated radiation therapy; RT = radiation therapy.

Table 2 lists the performance of 12 ML classifiers using 8 different algorithms in terms of each model's AUC, TPR (sensitivity), and TNR (specificity). Accuracy was biased strongly toward the majority negative class ($Desq^{-})$ as shown by consistently high TNRs and low TPRs across all models, likely due to class imbalance in the ITD. The 3 best-performing classifiers in terms of AUC in the VD were LMT, RF, and NB with 0.75, 0.74, and 0.74, respectively. These were selected for cost-sensitive learning optimisation with incremental penalty rising in 10 steps from 10 to 100. All 12 ML classifiers listed in Table 1 were also applied to the 3 resampled training data sets (RUS, ROS, and SMOTE).

**Table 2:** Model performance with imputed imbalanced training data set DMI(ITD) and validation data set DMI(VD)

	Training in ITD (n = 1029)			Validation in VD (n = 1029)			
Classifier	Specificity (TNR)	Sensitivity (TPR)	AUC	Specificity (TNR)	Sensitivity (TPR)	AUC	Rank
(K = 1) NN	0.908	0.167	0.548	0.923	0.292	0.607	9
(K = 3) NN	0.975	0.094	0.601	0.979	0.125	0.627	8
(K = 5) NN	0.985	0.042	0.624	0.989	0.063	0.651	6
(K = 7) NN	0.996	0.031	0.648	0.998	0.052	0.644	7
(K = 9) NN	0.999	0.031	0.660	0.999	0.042	0.665	5
ANN	0.945	0.198	0.694	0.953	0.177	0.676	4
C4.5	0.985	0.083	0.575	0.979	0.125	0.496	12
LMT	0.996	0.010	0.578	0.995	0.042	0.746	1
LR	0.910	0.188	0.567	0.959	0.135	0.596	10
NB	0.810	0.438	0.697	0.833	0.500	0.737	3
SVM	0.966	0.156	0.561	0.976	0.146	0.561	11
RF	0.998	0.021	0.725	0.999	0.010	0.742	2

Abbreviations: ANN = artificial neural network; AUC = area under the curve; C4.5 = decision tree; DMI = decision-tree based missing value imputation; ITD = imbalanced training; KNN = K-nearest neighbor; LMT = logistic model tree; LR = logistic regression; NB = naïve Bayes; RF = random forest; SVM = support vector machine; TNR = true negative rate; TPR = true positive rate; VD = validation.

**Fig. 2:**
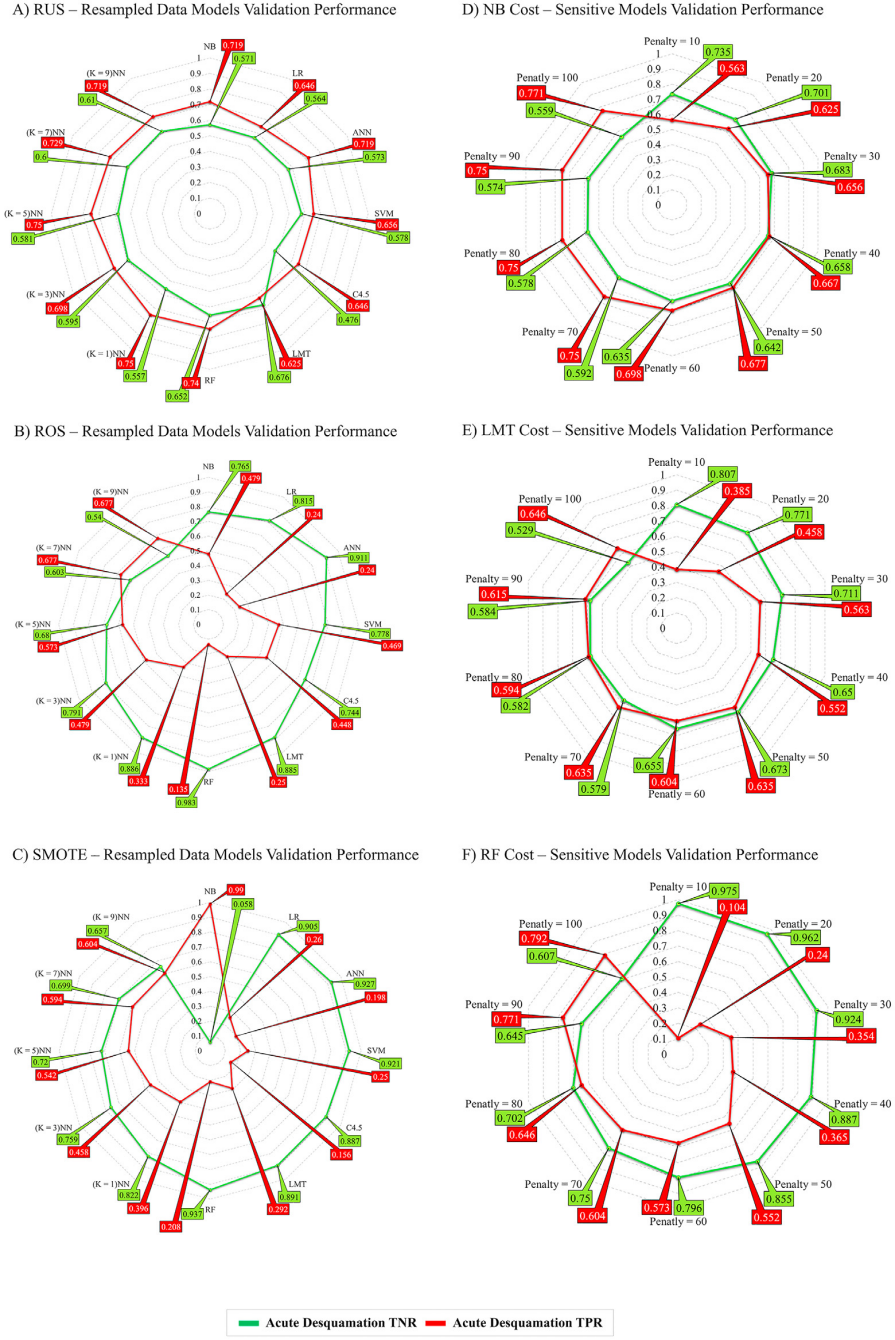
Radar charts plotting sensitivity (TPR) and specificity (TNR) in the validation data set for all ML models developed with the RUS, ROS, and SMOTE resampled training data and after applying cost-sensitive learning to the 3 best-performing ML models (RF, NB, and LMT). Abbreviations: LMT = logistic model tree; ML = machine learning; NB = naïve Bayes; RF = random forest; ROS = random over-sampling; RUS = random under-sampling; SMOTE = synthetic minority oversampling technique; TNR = true negative rate; TPR = true positive rate.

Figure 2 shows radar charts plotting sensitivity (TPR) and specificity (TNR) in the VD for a total of 66 models in the resampled training data (Fig. 2A-C) and after applying cost-sensitive penalties to the 3 best performing classifiers (Fig. 2D-F). Resampling improved sensitivity across all classifiers, with RUS (Fig. 2A) achieving the least variance between specificity and sensitivity on validation. For the cost-sensitive classifiers, the incremental penalty skewed the correct classification toward the true positives and models with higher penalty showed higher sensitivity (TPR). NB model sensitivity ranged from 0.50 in the unpenalized model to 0.77 for a penalty of 100. The largest improvement in sensitivity was achieved for the RF classifier, ranging from 0.01 for the unpenalized model to 0.79 at penalty of 100. LMT sensitivity improved from 0.04 without a penalty to 0.65 with a penalty of 100. Specificity (TNR) decreased for all 3 cost-sensitive classifiers because the number of predicted false-positives increased with each incremental penalty.

### Model selection and feature filtering

**Fig. 3:**
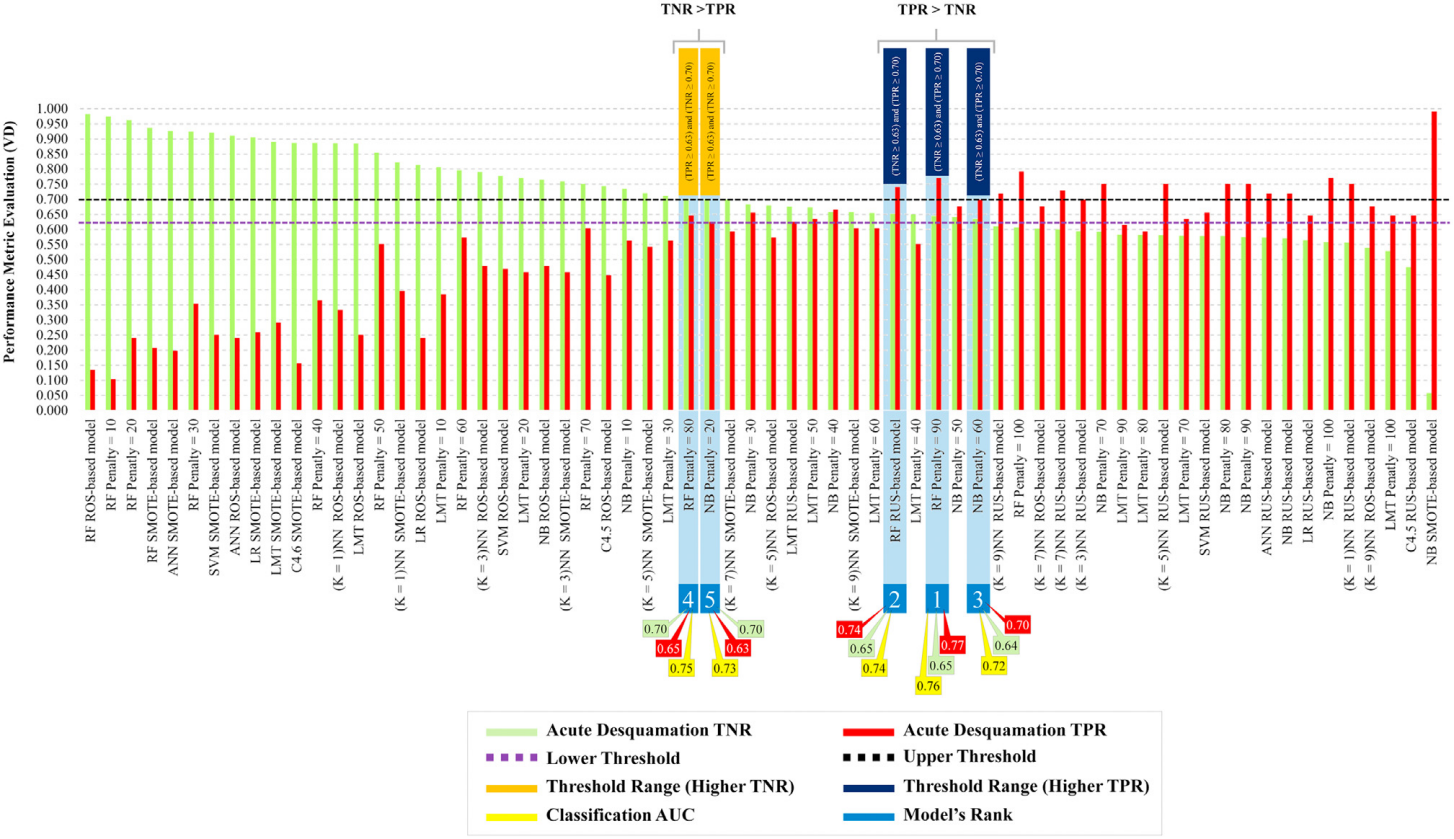
Trade-off threshold lines are shown for sensitivity (TPR) and specificity (TNR) at 0.63 and 0.70, respectively. Five models cross both threshold lines and their TPR, TNR, and AUC values are shown at the bottom. Two out of 5 models have a higher TNR than TPR and 3 out of the 5 models have a higher TPR than TNR. The “hero” model (no. 1) was the cost-sensitive random forest algorithm with a penalty of 90:1. *Abbreviations:* AUC = area under the curve; TNR = true negative rate; TPR = true positive rate.

Figure 3 shows 2 conditions (finishing lines) selected to maximize accuracy, that is, maximizing both TPR and TNR (the clinicians’ trade-off), with lower and upper threshold values of 0.63 and 0.70, respectively, which were crossed by 5 classifiers: cost-sensitive RF (CS-RF) with an FN:false positive (FP) 90:1 penalty (TNR = 0.65, TPR = 0.77, AUC = 0.76); RUS-RF (TNR = 0.65, TPR = 0.74, AUC = 0.74); cost-sensitive NB with an FN:FP 60:1 penalty (TNR = 0.64, TPR = 0.70, AUC = 0.72); CS-RF with an FN:FP 80:1 penalty (TNR = 0.70, TPR = 0.65, AUC = 0.75); and cost-sensitive NB with an FN:FP 20:1 penalty (TNR = 0.70, TPR = 0.63, AUC = 0.73). As maximizing sensitivity (TPR) was most important, the best performing “hero” model was the CS-RF classifier with an FN:FP penalty of 90:1. This model exceeded others for sensitivity and AUC performance while maintaining moderate specificity.

The hero CS-RF (90:1) model had m = 122 features. Eight features were estimated to have zero importance including features about presence/absence of systemic lupus erythematosus and other collagen vascular diseases and use of pertuzumab, eribulin, and amiodarone therapy. In a final step, these features were removed and the model was rebuilt and revalidated in the VD. Table 3 lists the features included in the final hero CS-RF classifier by order of importance. In descending order, the top 10 features were duration of other lipid-lowering drug use, type of surgery (wide local excision vs quadrantectomy), use of radiation therapy bolus, use of chemotherapy, use of boost, radiation therapy photon dose (MV), use of epirubicin therapy, hypertension, bra band size, and side of radiation therapy. Performance of the optimized hero final model in the VD improved slightly in terms of specificity (TNR = 0.66) and AUC (0.77) while sensitivity (TPR) remained unchanged.

**Table 3:** Features in the “hero” optimized cost-sensitive RF classifier ranked by importance

Model's feature	MDI	Model's feature	MDI
other_lipid_lowering_drugs_duration_yrs	0.52	alcohol_current_consumption	0.2
surgery_type	0.41	smoking_time_since_quitting_yrs	0.2
radio_bolus	0.4	radio_imrt	0.19
chemotherapy	0.36	radio_photon_boostdose_Gy	0.19
boost	0.35	other_antihypertensive_drug	0.19
radio_photon_dose_MV	0.34	household_members	0.19
epirubicin_chemo_drug	0.34	radio_breast_fractions_dose_per_fraction_Gy	0.19
blood_pressure	0.33	radio_elec_boost_field_y_cm	0.19
Bra_band_size	0.3	radio_photon_2nd	0.19
radio_treated_breast	0.3	bra_cup_size	0.19
tumour_size_mm	0.29	radio_breast_fractions	0.19
paclitaxel_chemo_drug	0.29	n_stage	0.18
grade_invasive	0.28	hypertension_duration_yrs	0.18
breast_separation	0.28	radio_supraclavicular_fossa	0.18
smoking	0.27	education_profession	0.18
radio_elec_energy_MeV	0.27	radio_axillary_levels	0.18
BED_boost	0.27	hypertension	0.18
docetaxel_chemo_drug	0.27	radio_photon_boost_fractions_per_week	0.17
BED_Total	0.27	smoker	0.17
radio_elec_boost_dose_Gy	0.27	depression	0.17
On_tamoxifen	0.26	menopausal_status	0.17
radio_heart_mean_dose_Gy	0.26	radio_boost_diameter_cm	0.16
t_stage	0.26	5-fluorouracil (5-FU)_chemo_drug	0.16
radio_hot_spots_107	0.25	radio_photon_boost_dose_per_fraction_Gy	0.16
BED_Breast	0.25	antidepressant_duration_yrs	0.16
tobacco_products_per_day	0.25	radio_breast_fractions_per_week	0.15
age_at_radiotherapy_start_yrs	0.25	radio_boost_type	0.15
radio_breast_ct_volume_cm3	0.25	Carboplatin_chemo_drug	0.15
hormone_replacement_therapy	0.24	radio_boost_sequence	0.15
radio_photon_boost_volume_cm3	0.24	radio_photon_boost_fractions	0.15
antidepressant	0.24	household_income	0.15
height_cm	0.24	methotrexate_chemo_drug	0.15
radio_photon_2nd_energy_MV	0.24	other_lipid_lowering_drugs	0.14
radio_ipsilateral_lung_mean_Gy	0.24	radio_photon_energy_MV or kV	0.14
alcohol_previous_consumption	0.24	ace_inhibitor	0.13
radio_photon_2nd_dose_fractions_per_week	0.23	analgesics_duration_yrs	0.13
radio_skin_max_dose_Gy	0.23	radio_photon_2nd_dose_per_fraction_Gy	0.13
histology	0.23	antidiabetic_duration_yrs	0.13
monopause_age_yrs	0.23	depression_duration_yrs	0.13
other_antihypertensive_drug_duration_yrs	0.23	on_statin_duration_yrs	0.12
weight_at_cancer_diagnosis_kg	0.23	antidiabetic	0.12
tobacco_product	0.23	diabetes	0.11
cyclophosphamide_chemo_drug	0.22	ace_inhibitor_duration_yrs	0.11
combined_chemo_drugs	0.22	on_statin	0.11
boost_frac	0.22	doxorubicin_chemo_drug	0.11
analgesics	0.22	history_of_heart_disease	0.09
breast_cancer_family_history_1st_degree	0.22	radio_axillary_other	0.09
smoking_duration_yrs	0.21	ethnicity	0.09
radio_photon_boostdose_precise_Gy	0.21	radio_interrupted	0.08
radio_elec_boost_field_x_cm	0.21	pegfilgrastim_chemo_drug	0.07
radio_photon_2nd_fractions	0.21	history_of_heart_disease_duration_yrs	0.06
radio_boost_fractions	0.21	radiotherapy_toxicity_family_history	0.06
alcohol_intake	0.21	diabetes_duration_yrs	0.05
radio_type_imrt	0.21	radio_interrupted_days	0.05
radio_treatment_pos	0.21	trastuzumab_chemo_drug	0.04
radio_breast_dose_Gy	0.2	other_collagen_vascular_disease	0.03
rheumatoid arthritis_duration_yrs	0.2	rheumatoid arthritis	0.02

*Abbreviations:* BED = biologically effective dose; IMRT = intensity modulated radiation therapy; MDI = mean decrease impurity; MeV = mega electron volt; MV = mega volt; RF = random forest*.*

Feature importance is calculated as the decrease in node impurity weighted by the probability of reaching that node. The node probability can be calculated by the number of samples that reach the node, divided by the total number of samples. The higher the value, the more important the feature.

## Discussion

A recently published logistic regression model for acute breast desquamation after adjuvant external beam breast radiation therapy developed in 3 combined external breast radiation therapy cohorts failed to validate in the multicenter REQUITE cohort.^24^ The aim of this study was to use ML algorithms to develop and optimise a prediction model for acute desquamation in the REQUITE breast cancer cohort . ML techniques have previously been used to predict acute skin toxicity during breast radiation therapy.^22^^,^^23^ We elected to predict the occurrence of acute desquamation rather than dermatitis (skin erythema) because it can cause clinically significant patient morbidity and can worsen the cosmetic outcome after breast surgery. This accounts for the lower proportion of cases with skin toxicity reported in our study versus the study by Saednia et al^22^ (0.09 vs 0.38), although the proportion of cases was similar to those with moist desquamation in the abstract published by Reddy et al.^23^

Predicting cases of clinically significant radiation toxicity such as acute desquamation remains challenging for both parametric statistical and ML models due to the issue of class imbalance leading to high FN rates, that is, poor sensitivity. In this study, a combination of resampling techniques and cost-sensitive learning was used to try and improve predictive performance. RUS and cost-sensitive optimisation contributed the most to optimal performance across the different ML algorithms. Of 66 models tested, 5 fulfilled prespecified criteria for maximizing both TPR and TNR. On the basis of highest TPR, the hero model was the CS-RF, with an FN:FP misclassification penalty of 90:1. Given that our modeling used somewhat fewer features and had a multicenter patient sample with diverse radiation treatment regimens, it is reassuring that its AUC of 0.77 in the VD is similar to the range of AUCs reported in the abstract by Reddy et al.^23^

Our initial models for acute desquamation included 122 features. Information gain (IG) represents the amount of information gained about a random variable or signal from observing another random variable. After the randomized and stratified training/validation data split, only a few variables in the VD had a different IG to discriminate between the positive and negative cases compared with the ITD. Zero IG does not negate the feature's worth as this depends on the ML algorithm used, and any given feature could climb up the ranking in terms of IG if additional observations were added to the same data set. Hence, we included all 122 features in the modeling process. The 10 most important features in the final hero model included some that might be expected to predict breast radiation toxicity, such as use of radiation therapy bolus, chemotherapy, boost, radiation therapy dose, and bra size. Interestingly, the most important feature (use of lipid-lowering drugs) is not usually included in parametric statistical models for radiation toxicity, although HMG-CoA reductase inhibitors (statins) have previously been proposed as radioprotective agents.^50^ Yet unlike traditional statistical probability modeling, feature importance should only be interpreted within the context of the ML prediction model but not outside.

### Study limitations

Despite the rigorous error detection in the data preprocessing phase, we cannot exclude errors occurring due to manual recording during data collection. According to the REQUITE study protocol, patients were assessed at the start and end of treatment and annually thereafter. This may have missed cases of acute desquamation as acute radiation toxicity is known to peak up to 2 weeks after the end of treatment. Although we incorporated differences in radiation therapy techniques by including all available recorded treatment parameters in the analysis, this may not fully account for variability in treatment plans between participating centers or treating physicians. Similarly, variable transformation or feature engineering (eg, calculating the BED, binarization of chemotherapy drugs) could have led to the creation of a new feature that is less powerful and suppresses important information inferred by its raw components. In modeling the radiation therapy dose variable, alternatives such as a categorical variable divided by type of radiation therapy regimen could have been used (eg, hypo- vs standard fractionation). Variable aggregation could have led to model overfitting due to misleading combined features and may show false significance or insignificance in the analysis.^51^ Although the resampling techniques used in this study have advantages in their simplicity and transportability, other remedies to address imbalanced data, such as ensemble learning (which is implemented at the algorithmic level), could be used to improve model performance.^52^ Cost-sensitive learning was selected to penalize false negatives. However, its application depends on the clinical situation. For example, if a model was designed to allocate patients to a toxicity-lowering radiation therapy regimen that might affect tumor control, then FPs may need to have a higher cost than FNs. This study used the impurity-based ranking mean decrease impurity filter to simplify the final model with a known performance, but it is important to keep in mind that feature selection based on impurity reduction is generally biased toward preferring variables with more categories.^53^

## Conclusion

Application of ML algorithms with resampling and cost-sensitive learning resulted in valid prediction models for acute desquamation after whole breast EBRT using clinical and treatment features. After optimisation, the best model was able to classify patients with acceptable performance in the validation cohort (AUC = 0.77). Before they can be used in clinical practice, further optimization of ML prediction models, including genomic markers, is required, and the models should be validated in external cohorts. This approach could help identify breast cancer patients at increased risk of toxicity to inform discussions about risks and benefits and allow treatment plans to be personalized with the aim of minimizing toxicity or offering the patient increased supportive management during treatment.

## Appendix Supplementary materials

Image, image 1

Image, image 2
