# Supplementary figures and images for: Development and Optimization of a Machine-Learning Prediction Model for Acute Desquamation After Breast Radiation Therapy in the Multicenter REQUITE Cohort
Source: Adv Radiat Oncol. 2022 Jan 3;7(3):100890. doi: 10.1016/j.adro.2021.100890 (PMC9133391; doi:10.1016/j.adro.2021.100890)

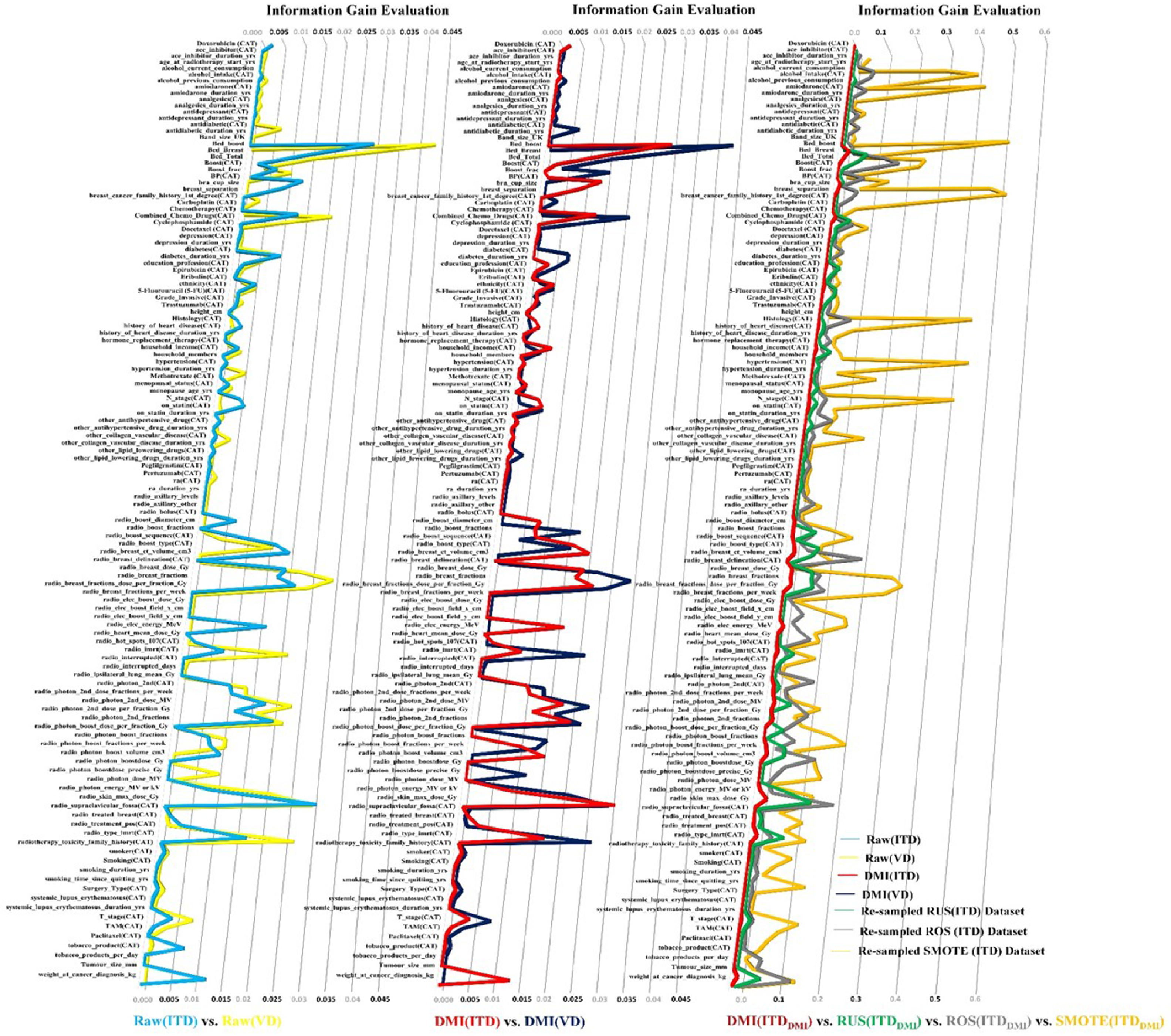

Supplement: Supplementary file 1 [file mmc1.jpg]

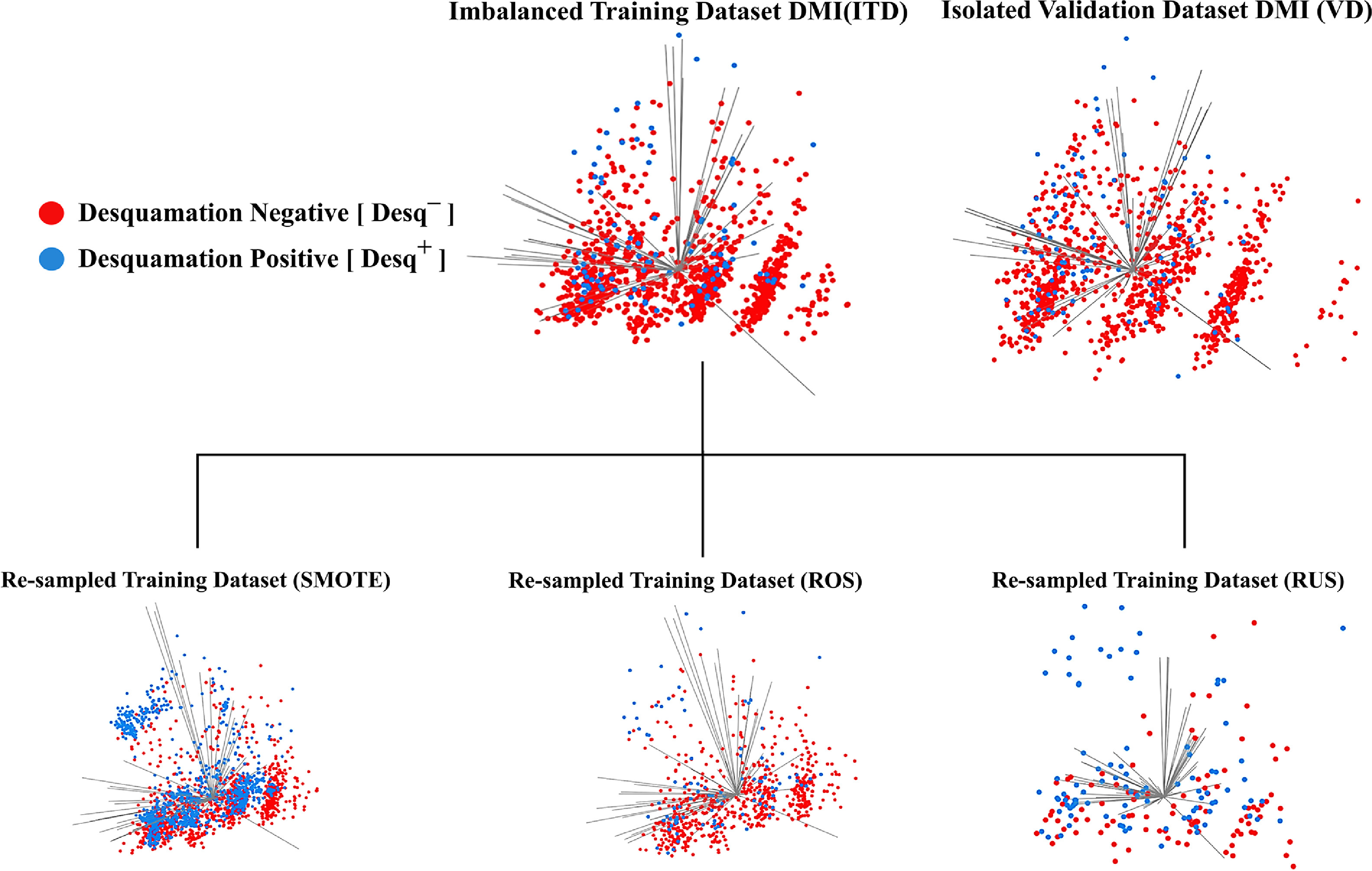

Supplement: Supplementary file 2 [file mmc2.jpg]
